# Organic light-emitting diode-based photodynamic therapy treats bacterial infection in a preclinical *ex vivo* burn wound model

**DOI:** 10.1093/burnst/tkag024

**Published:** 2026-03-31

**Authors:** Vincent De Maesschalck, Mina Riahi, Rob Lavigne, Greetje Vande Velde, Ifor D W Samuel

**Affiliations:** Laboratory of Gene Technology, Department of Biosystems, KU Leuven, Kasteelpark Arenberg 20 Box 2462, 3001 Leuven, Belgium; Organic Semiconductor Centre, School of Physics and Astronomy, University of St Andrews, North Haugh, St Andrews, Fife KY16 9SS, United Kingdom; Laboratory of Gene Technology, Department of Biosystems, KU Leuven, Kasteelpark Arenberg 20 Box 2462, 3001 Leuven, Belgium; Biomedical MRI, Department of Imaging and Pathology, KU Leuven, Herestraat 49, Box 505, 3000 Leuven, Belgium; Organic Semiconductor Centre, School of Physics and Astronomy, University of St Andrews, North Haugh, St Andrews, Fife KY16 9SS, United Kingdom

**To the Editor,**

The global rise of antimicrobial resistance has created an urgent need for alternatives to conventional antibiotics. One emerging approach is photodynamic therapy (PDT) [1], which uses light-activated photosensitizers to generate reactive oxygen species (ROS) that kill microorganisms. Although PDT is already used in dermatology, and is typically delivered using bulky lasers or lamps, more practical light sources are needed for real-world antimicrobial applications [2]. Organic light-emitting diodes (OLEDs) offer a promising solution: they are thin, flexible, wearable, and inexpensive, making them compatible with bandage-like therapeutic devices [3].

A previous study [4] demonstrated that flexible OLEDs can support antimicrobial PDT *in vitro*, but translation to clinically relevant scenarios has been lacking. In this letter, OLED-PDT is tested in a porcine *ex vivo* burn wound model, which closely resembles human skin. The model was infected with a clinically relevant, penicillin-resistant Staphylococcus aureus strain, which is a major opportunistic pathogen that frequently colonizes burn wounds. To enable the real-time monitoring of bacterial proliferation, the S. aureus strain carried a lux operon [5], allowing bioluminescence to serve as a quantitative proxy for the bacterial burden.

The custom OLED used in these experiments delivered an irradiance of 9.62 mW/cm^2^ at a current density of 25 mA/cm^2^ with an operating voltage of 3.92 V, emitting at a peak wavelength of 622 nm. To reduce the operating voltage and hence Joule heating, the charge transport layers were doped to make a p-i-n structure (consisting of a p doped hole transport layer and an n doped electron transport layer). Additional characteristics of the OLED can be found in Supplementary Figure S1.

*Ex vivo* porcine skin samples (~1 × 1 cm) were excised and positioned on agar within a 24-well plate to maintain tissue hydration. Circular burn wounds were generated using a heated metal rod and then inoculated with 10^5^ CFU of S. aureus Xen36. After a 3-h incubation at 37°C to allow for attachment and early growth, the explants entered the treatment phase. Half of the OLED-exposed samples received 20 μg of methylene blue (MB) as the photosensitizer, while the remaining samples received buffer only. Additional infected and uninfected controls were also included.

The experimental setup is illustrated in Figure 1a and b, showing the OLED panel above the well plate, illuminating five of the six columns, while column six remained unilluminated. Bioluminescence was measured every 30 min over 4 h, generating longitudinal bacterial growth curves.

**Figure 1:**
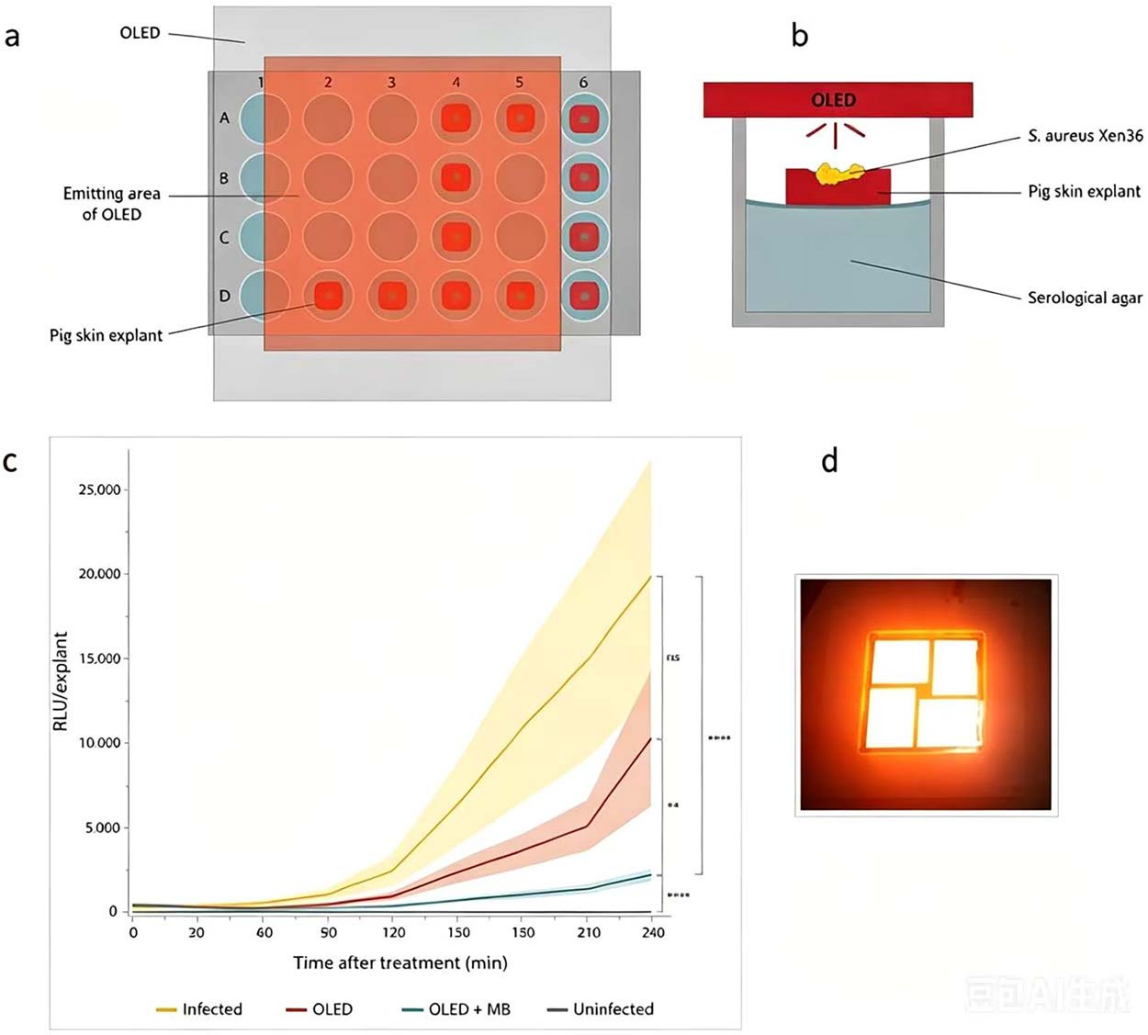
Experimental set-up of the *ex vivo* antibacterial OLED-PDT model. (**a** and **b**) Pig skin explants of approximately 1 × 1 cm carrying a circular burn wound were each placed on top of agar in the individual wells of a 24-well plate, allowing the OLED to be placed on top of this plate during the treatment phase of the experiment. The OLED device is schematically represented as a large square above the well plate. (**c**) Antibacterial effect of OLED-PDT (i.e. OLED+methylene blue (MB)) in an *ex vivo* burn wound model. (**d**) Photograph of the OLED devices used in the experiment. *OLEDs* organic light-emitting diodes, *PDT* photodynamic therapy

After a 3-h bacterial attachment phase, explants were treated with 20 μg of MB, followed by 4 h of OLED illumination. Longitudinal bioluminescence measurements revealed that OLED-PDT significantly suppressed bacterial growth compared to both untreated and OLED-only controls. As shown in Figure 1c, log-transformed relative light units (RLU) values showed a 78.5% reduction in bacterial burden after 4 h relative to OLED-only treatment (*P* = .0082) and a highly significant reduction compared to untreated infected samples (*P* < .0001). OLED illumination without a photosensitizer did not significantly reduce bacterial growth (*P* = .2120). Bioluminescence intensity measurements, expressed in RLU per explant, correlated with bacterial burden (Supplementary Figure S2). A graph showing all individual data points is available in Supplementary Figure S3. Error bands were created using one standard error from the mean. The Dunnett’s *post hoc* analysis provided in Supplementary Table S1 further indicated that bacterial reduction became significant after 90 min of treatment, demonstrating a rapid onset of antimicrobial action.

While OLED-PDT effectively reduced the bacterial load by nearly one order of magnitude, the bioluminescence signal remained above the uninfected baseline, indicating that a single round of treatment under this specific regimen did not achieve complete sterilization. Temperature monitoring provided in Supplementary Figures S4 and S5 confirmed limited heating of the explants, supporting the safety of prolonged OLED use in close proximity to tissue.

Overall, combining OLED illumination with MB achieved an approximately 80%–88% reduction in bacterial burden, demonstrating substantial antibacterial activity in a clinically relevant *ex vivo* environment.

OLED-PDT holds several advantages over conventional antibiotics and even over other PDT delivery systems. Unlike antibiotics, which require complex delivery strategies and face increasing rates of resistance, the OLED-PDT approach utilizes a straightforward topical application of MB that is already used clinically and light is delivered via a thin, low-voltage and potentially flexible device. PDT generates ROS through light activation, which differs from the primary mechanisms of antibiotics. This difference enables potential synergy in combination therapies and reduces concerns regarding cross-resistance. Although bacteria can in principle evolve enhanced tolerance to oxidative stress, previous work has failed to generate OLED-PDT-resistant S. aureus strains, supporting its suitability as a sustainable antimicrobial strategy.

The *ex vivo* model’s longitudinal readout capabilities allow for a detailed analysis of treatment kinetics and provide a valuable platform for optimizing dosing, treatment duration, and photosensitizer application patterns critical steps before *in vivo* translation. Future studies may explore different MB concentrations, repeated dosing schedules, and combinations with antibiotics. Given PDT’s antimicrobial breadth, OLED-PDT may also be applicable to fungal coinfections common in chronic wounds.

This study provides the first demonstration of OLED-based PDT in a clinically relevant *ex vivo* burn wound model, confirming significant antibacterial efficacy against multidrug-resistant S. aureus. OLEDs achieved effective PDT while maintaining safe temperature profiles, supporting their potential translation into wearable therapeutic devices, such as smart bandages, for ambulatory antimicrobial treatment.

## Supplementary Material

Supplementary_Information_final_tkag024

## Data Availability

The research underpinning this publication can be accessed at: https://doi.org/10.17630/fbdb65d7-636e-4b17-8cfe-e82eb568632c
