# Supplementary material for: Organic light-emitting diode-based photodynamic therapy treats bacterial infection in a preclinical ex vivo burn wound model
Source: Burns Trauma. 2026 Mar 31;14:tkag024. doi: 10.1093/burnst/tkag024 (PMC13313526; doi:10.1093/burnst/tkag024)
Supplement: Supplementary_Information_final_tkag024 [file supplementary_information_final_tkag024.docx]

**Supplementary information**


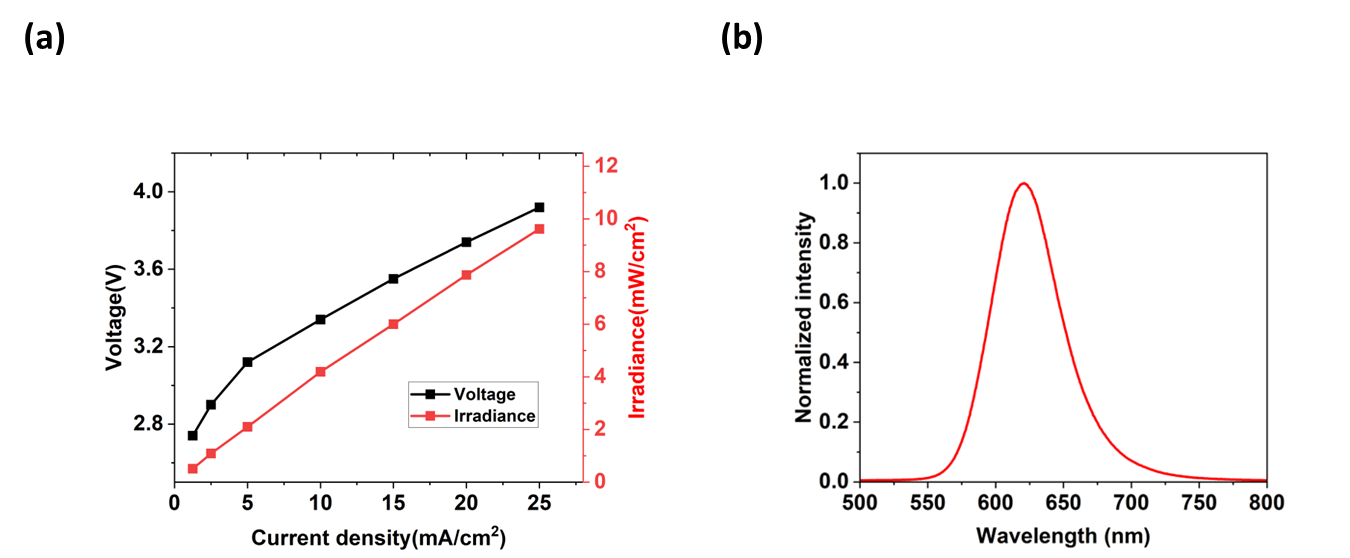


**Figure S1: Voltage-current density-irradiance characteristics and emission spectrum of constructed OLEDs used in this work.** Figure S1a shows the voltage-current density-irradiance characteristics of OLEDs as measured with a source measurement unit (Keithley 2400, Keithley, Cleveland, OH, USA) and an optometer (P9710, Gigahertz Optik, Türkenfeld, Germany). At a current density of 25 mA/cm^2^, OLED devices deliver an irradiance of 9.62 mA/cm^2^ with an operating voltage of 3.92 V, allowing to use a simple power supply to drive the OLED. Figure S1b shows the electroluminescence spectrum of the OLEDs with a peak wavelength of 622 nm as measured by a spectrograph (MS125, Oriel Instruments, Irvine, CA, USA) coupled to a charge-coupled device (CCD) camera (DV420-BU, Andor, Belfast, UK).

**Figure S2: Linear correlation between bacterial load and the bioluminescence intensity of *S. aureus* Xen36.** A linear relation between the log transformations of both bioluminescent signal (RLU/s/explant) obtained from the explant and bacterial number (CFU/explant) was obtained, with an R^2^ of 0.9598. This confirms a linear relationship between bioluminescence obtained from the explant and bacterial burden. Each time point consists of three biological replicates (n = 3). The error bars represent one standard deviation from the mean.


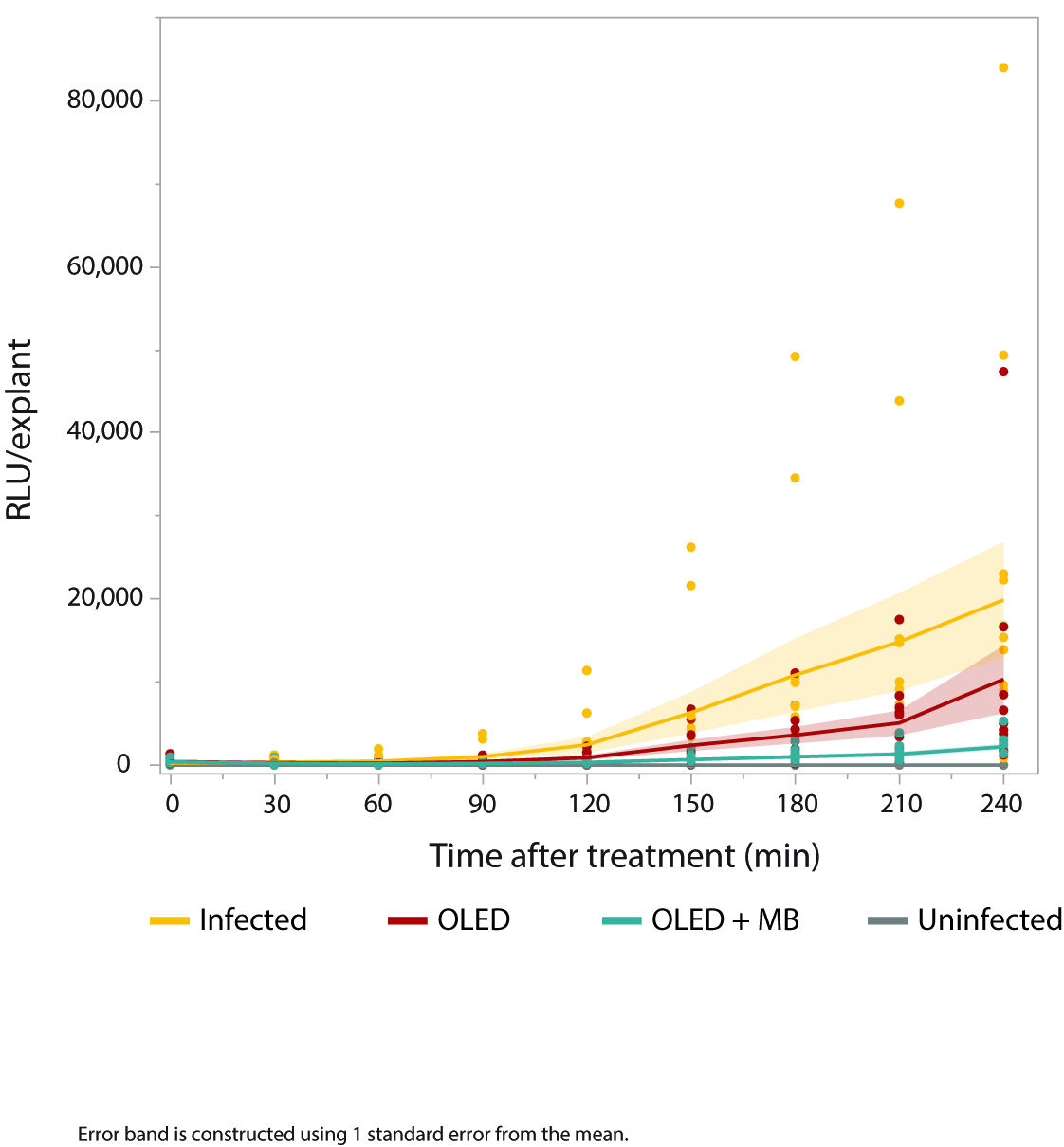


**Figure S3: Antibacterial effect of OLED-PDT in an *ex vivo* burn wound model, with individual data points**. After three hours of growth at 37°C, half of the OLED-exposed explants were treated with 20 µg methylene blue as a photosensitizer, whereas the other half was treated with buffer and all explants were exposed to the OLED. An infected and uninfected control was included as well. Error bands were created using one standard error from the mean.

**Table S1: A significant antibacterial effect ex vivo is visible after 90 minutes of treatment.** The p-values of Dunnett’s test with ‘infected’ condition as control condition. Starting from 90 minutes post treatment, a significant difference at significance level α = 0.05 is visible. Over all measured time points, a significant difference was observed between infected and uninfected tissue, meaning successful infection. On the other hand, no significant difference was observed between infected and OLED-illuminated tissue, suggesting no antibacterial effect of the OLED treatment alone. Significant conditions are highlighted in bold.

| *Time after treatment* | *Infected vs uninfected* | *Infected vs OLED* | *Infected vs OLED+MB* |
| --- | --- | --- | --- |
| 0 minutes | **<0.0001** | 0.3427 | 0.2786 |
| 30 minutes | **<0.0001** | 0.9298 | 0.5524 |
| 60 minutes | **<0.0001** | 0.3668 | 0.0688 |
| 90 minutes | **<0.0001** | 0.3999 | **0.0403** |
| 120 minutes | **<0.0001** | 0.4685 | **0.0217** |
| 150 minutes | **<0.0001** | 0.6124 | **0.0147** |
| 180 minutes | **<0.0001** | 0.5377 | **0.0095** |
| 210 minutes | **<0.0001** | 0.4307 | **0.0044** |
| 240 minutes | **<0.0001** | 0.5034 | **0.0026** |


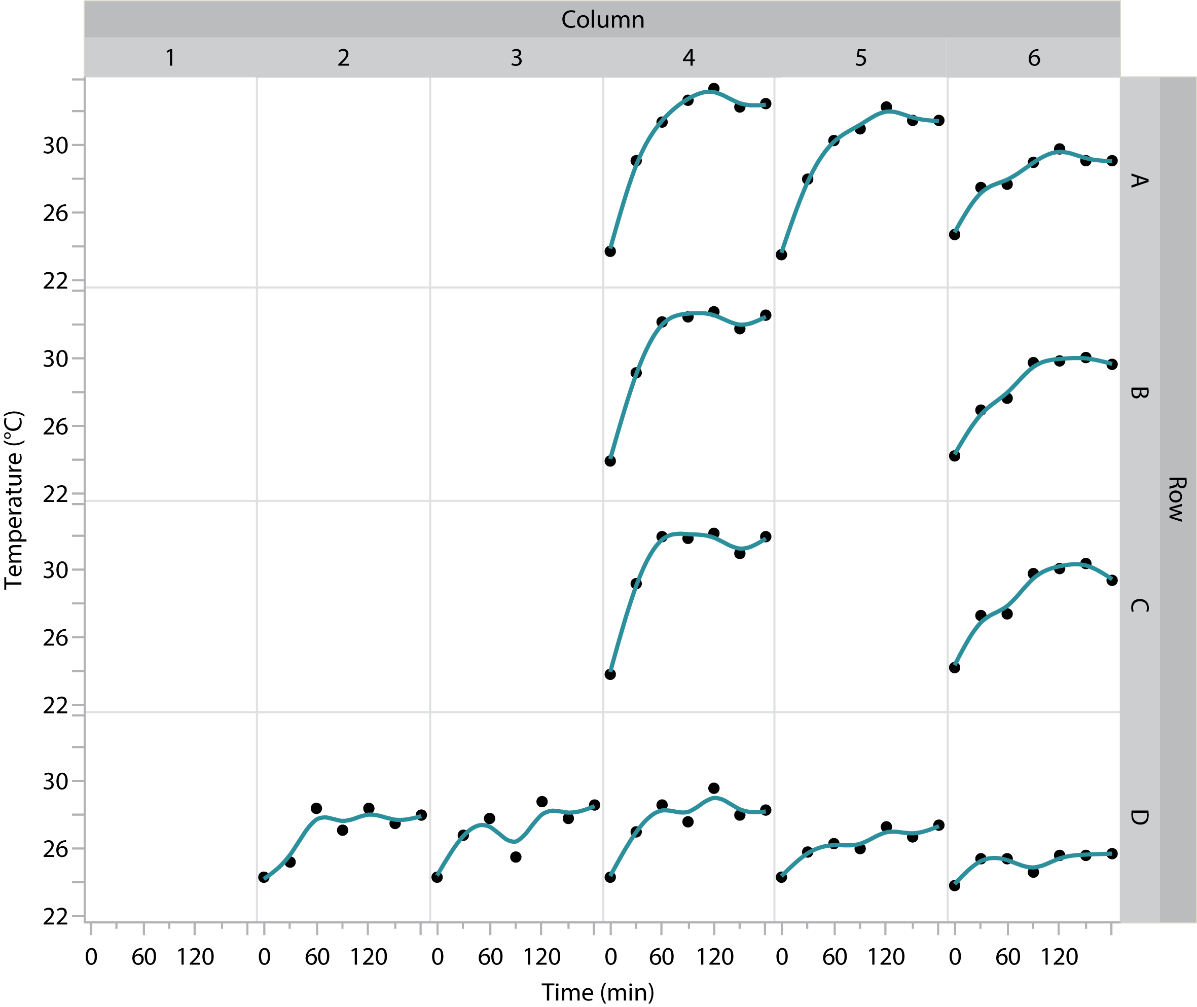


**Figure S4: Temperature profile of explants on room temperature exposed to the OLED.** The graph displays a 24-well plate containing explants as illustrated in Figure 1. For each well containing an explant, a temperature profile was established by measuring skin temperature at each timepoint with an infrared thermometer. Explants located in the middle of the 24-well plate and OLED absorbed more heat compared to the wells at the outside of both the OLED and the well plate.


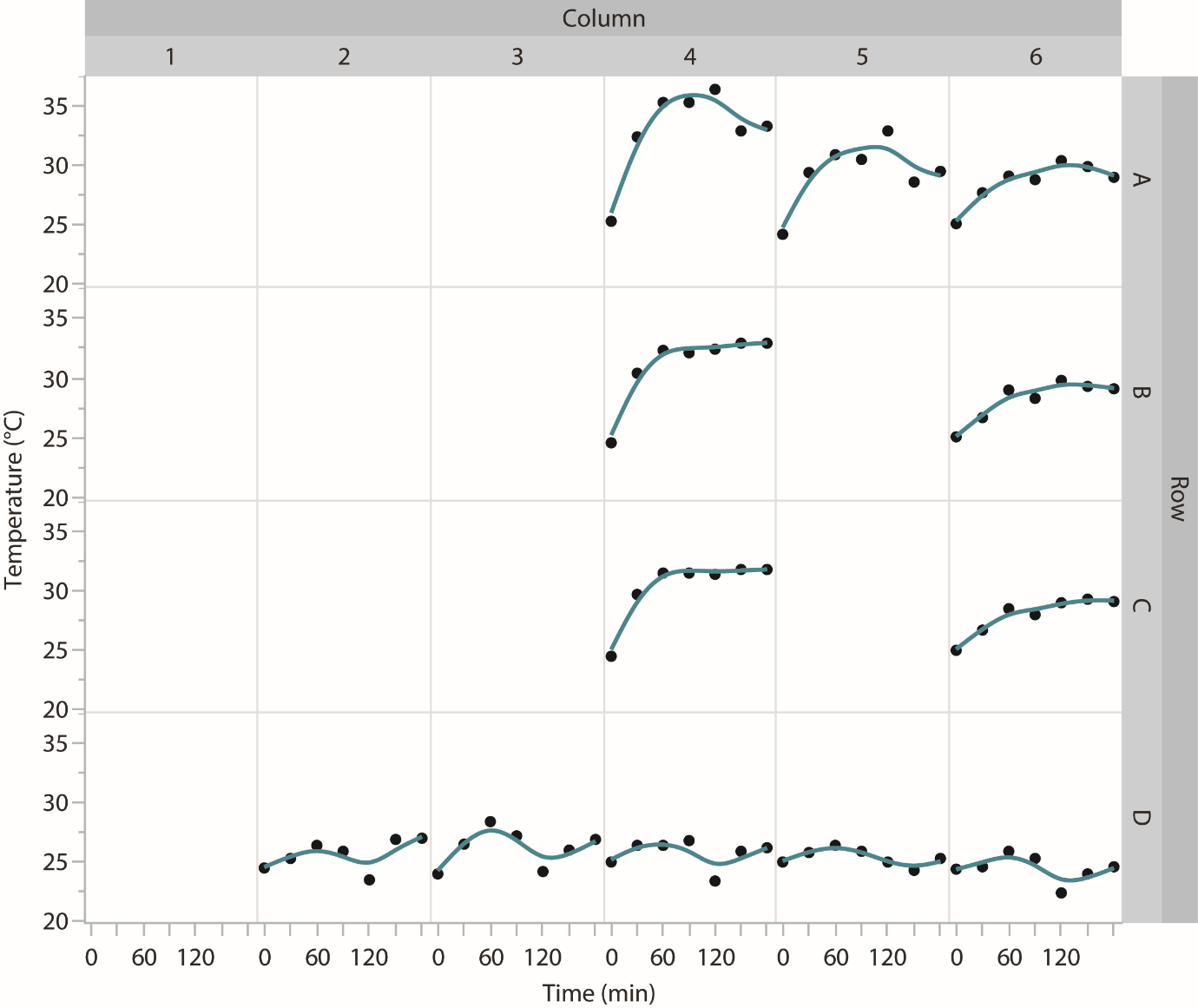


**Figure S5: Second biological replicate of the temperature profile of explants on room temperature exposed to the OLED**. The graph displays a 24 well plate containing explants as illustrated in Figure 1 of the manuscript. For each well containing an explant, a temperature profile was established by measuring skin temperature at each timepoint with an infrared thermometer. Explants located in the middle of the 24-well plate and OLED absorbed more heat compared to the wells at the outside of both the OLED and the well plate.
